# Histologic study of bone-forming capacity on polydeoxyribonucleotide combined with demineralized dentin matrix

**DOI:** 10.1186/s40902-016-0053-5

**Published:** 2016-02-13

**Authors:** Seok-Kon Kim, Chang-Kyu Huh, Jae-Hoon Lee, Kyung-Wook Kim, Moon-Young Kim

**Affiliations:** 1 Department of Pain and Anesthesiology, College of Medicine, Dankook University, 119 Dandae-ro, Dongnam-gu, Cheonan, Chungnam Korea; 2 Department of Oral and Maxillofacial Surgery, College of Dentistry, Dankook University, 119 Dandae-ro, Dongnam-gu, Cheonan, Chungnam Korea

**Keywords:** Polydeoxyribonucleotide (PDRN), Demineralized dentin matrix (DDM), Osteoinduction, Nude mice

## Abstract

**Background:**

This study examined the osteoinductive activity of demineralized dentin matrix (DDM) from human and polydeoxyribonucleotide (PDRN) for nude mice.

**Methods:**

Twenty healthy nude mice, weighing about 15~20 g, were used for the study. DDM from human and PDRN were prepared and implanted subcutaneously into the dorsal portion of the nude mice. The nude mice were sacrificed at 1, 2, and 4 weeks after grafting and evaluated histologically by hematoxylin-eosin and Masson’s trichrome staining. The specimens were also evaluated via a histomorphometric study.

**Results:**

The DDM and PDRN induced new bone, osteoblasts, and fibroblasts in soft tissues. The histological findings showed bone-forming cells like osteoblasts and fibroblasts at 1, 2, and 4 weeks. New bone formation was observed in the histomorphometric study. In particular, the ratio of new bone formation was the highest at 2 weeks compared with the first week and fourth week.

**Conclusions:**

In this study, we showed that the PDRN used in this experimental model was able to induce bone regeneration when combined to the DDM.

## Background

As an in vivo material for regeneration of tissue, polydeoxyribonucleotide (PDRN) is a DNA polymer with various different lengths used for wound healing and beauty through licenses as medicine in Europe, etc. [1].

It is known that if PDRN is used clinically, it has the function of shortening the healing period, normalizing tissues, and quick wound closure in diabetic foot ulcer, and is effective for contused wound and decubitus, wound healing, various scars, fine wrinkles, skin aging, skin photoaging, skin elasticity improvement, rapid healing after skin and plastic surgery procedures, alopecia, and blood circulation improvement [1]. Also, PDRN can be safely applied to a clinical setting because its activation disappears when it is orally administered and digested, and it is absorbed into the skin through injection and spread onto the skin and maintains activation even after high temperature sterilization [1].

Research on such special abilities of PDRN and trials to use it for treatment by working it on fibroblasts and promoting secretions of cell growth factor, as well as for beauty and wound healing, are active across the surgical field.

However, there has been a lack of academic studies or publishing on PDRN’s tissue regeneration function, especially bone generation, in the dental area where regeneration of bone tissue is essential, particularly in the oral and maxillofacial surgery, despite the development of various other graft materials.

In the oral and maxillofacial field, a lot of efforts have been made to form the bone more effectively, more rapidly, and in a more desired way for a more aesthetic regeneration of maxillary bone defects, along with the popularization of dental implants. In particular, human demineralized dentin matrix (DDM), announced through various research studies, includes multiple bone growth factors including type I collagen and BMP preserved in dentin, and thus helps in the rapid regeneration of alveolar bone and bone formation [2, 3]. Also, as it does not have a foreign body reaction and is biocompatible, it was used as a carrier to prevent the absorption of PDRN used in this study and to grant manipulability.

The purpose of the study was to jointly implant PDRN and DDM which was made from a human’s extracted tooth under the skin of the nude mice and investigate the expressed bone formation ability in a histological and histomorphometric way.

## Methods

### Research materials

#### Experimental animals

Twenty 4-week-old nude mice weighted about 15~20 g from Orient Bio Inc. (Seoul, Korea) were used as experimental animals and laboratory animals. After a 5-day adaptation period followed by confirmation of absence of natural abnormality, they were used for the experiment. They were raised with a free supply of water and solid feed in an environment of 22 °C average room temperature on a 12-h light-dark cycle. The policies of the Korean Association for Laboratory Animal Science and the laws related to animal experiments were observed.

#### Preparation of human DDM

A tooth extracted from a human was delivered in a state of being soaked in 70 % ethyl alcohol to a treating agency (Korea Tooth Bank Co., Seoul, Korea). Attached foreign substances such as soft tissue or dental calculus were removed, and then it was divided into crown and root and each part went through a crushing process. The 1~2-mm-sized crushed particles were put in distilled water and hydrogen oxide solution, and the remaining foreign substances were removed from it by washing with an ultrasonic cleaner. The washed particles were dehydrated with ethyl alcohol and went through defatting using an ethyl ether solution. The particles that completed all the processes up to this underwent lyophilization, and ethylene oxide gas sterilization was performed. Finally, the particles arrived at the laboratory in a packing state and were used for the implant process.

#### Preparation of PDRN

A 1.875 *w*/*v*% polydeoxyribonucleotide solution (Placentex Integro, Mastelli Srl, Sanremo, Italy) was prepared.

### Research methods

#### Subcutaneous implant

Pentobarbital sodium (Nembutal, 43 mg/kg, Dainabot Co., Japan) diluted in sterile water for injection was injected into the abdomen to induce general anesthesia, and then the surgical site was sterilized and isolated.

The dorsal portion was incised, and a subcutaneous pouch was formed in both sides. DDM, which was wet with 1.875 *w*/*v*% polydeoxyribonucleotide solution, 10 μg/ml, was implanted into the subcutaneous pouch, and the cut was sutured with nylon thread. And antibiotic ointment was applied to prevent postoperative infection.

#### Manufacturing and observation of tissue samples

Experimental animals were sacrificed at 1, 2, and 4 weeks, and DDM and neighboring tissue were immediately collected. The collected tissue was fixated in 10 % buffered formalin for more than 10 days. It was demineralized using formic acid and dehydrated with ethanol and embedded in paraffin to make the specimens. After hematoxylin-eosin and MT staining, the specimens were histopathologically observed with an Olympus BX-51 optical microscope (Olympus Co., Tokyo, Japan).

#### Histomorphometric analysis

The implanted PDRN causes an angiogenesis phenomenon in neighboring soft tissue and induces [4, 5] new blood vessels to the implant site and differentiates [6–8] undifferentiated mesenchymal cells of the host into fibroblast and osteoblast, and then differentiated bone formation cells form new bones such as osteoid. Therefore, to investigate the quantitative level of bone-forming cells around DDM, the value derived from dividing the number of bone-forming cells around dentin particles by the number of dentin particles was expressed as a graph (Fig. 6). Also, the specimens were augmented to the ×100 magnification of an optical microscope, and six points per week were randomly selected from the specimens at 1, 2, and 4 weeks and their digital image was obtained with a digital camera attached to the microscope. And then NB%, the area of newly formed mineralized bone to obtained image area, was calculated and analyzed using an image analysis program (Kappa Image Base-metreo, Opto-Electronics, Gleichen, Germany).

## Results

### Histological observation

**Fig. 1:**
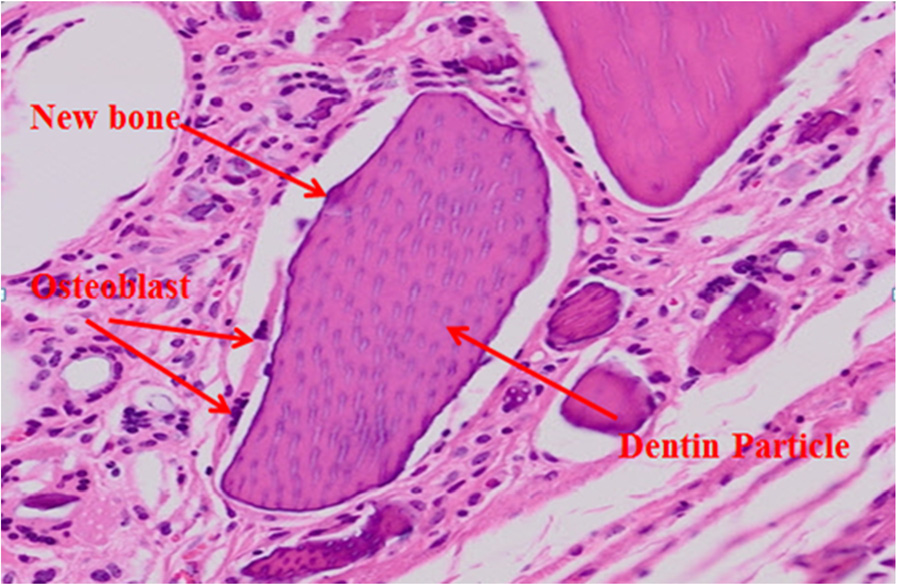
Histologic finding of DDM with PDRN after 1 week (H-E stain, ×500). Dense fibrous tissues with moderate blood vessels and newly attached osteoblast were observed around dentin particles

**Fig. 2:**
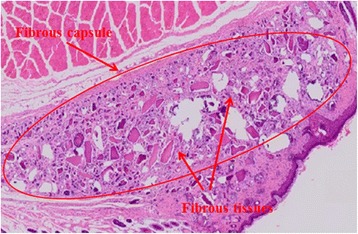
Histologic finding of DDM with PDRN after 1 week (H-E stain, ×100). Well-organized fibrous capsules are seen around the whole grafted materials. This showed little voids in the central area of grafted materials where it is difficult for the blood vessels to invade. The fibrous capsules around each particle are relatively tight and intimated

As for histopathological findings, a fibrous capsule which was well-bounded with neighboring tissue was observed to be surrounding the implant material and voids between implant materials and fibrous soft tissue, which played an important role in supplying the blood vessels and cells, were well developed. In addition, the vicinity of dentin particles with well-developed dentinal tubules was surrounded by fibroblast and osteoblast, where new bone being slightly formed was observed (Figs. 1 and 2).

**Fig. 3:**
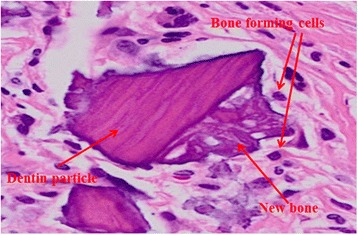
Histologic finding of DDM with PDRN after 2 weeks (H-E xstain, ×500). The finding showed fibroblast-like cells before phenotypic transformation and activated osteoblast-like cells

**Fig. 4:**
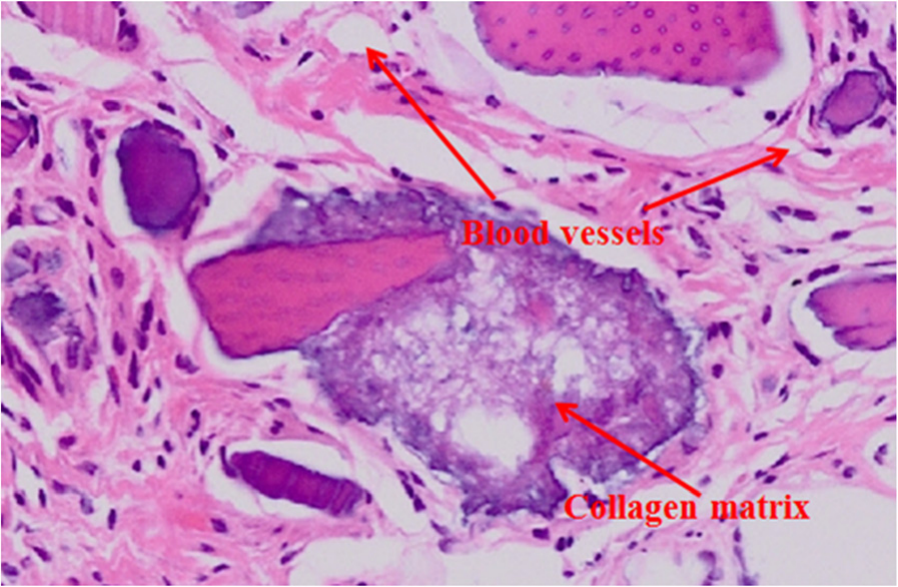
Histologic finding of DDM with PDRN after 2 weeks (H-E stain, ×500). Newly deposited and produced collagen matrix on dentin particles was observed

Much greater bone-forming cells were observed in the second week than in the first week, and development of the blood vessels and newly formed collagen matrix were observed between soft tissues surrounding dentin particles (Figs. 3 and 4).

**Fig. 5:**
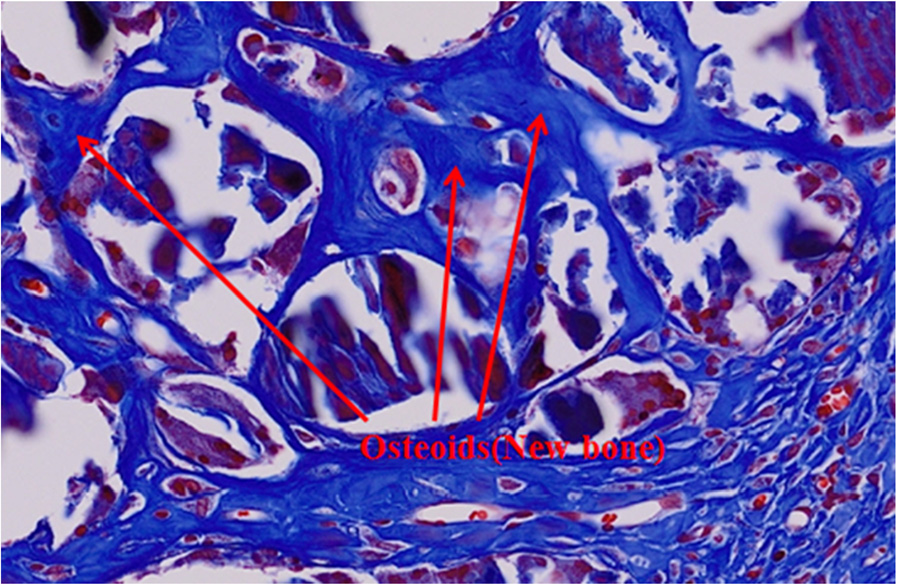
Histologic finding of DDM with PDRN after 4 weeks (MT-stain, ×500). The amount of new osteoid increased remarkably at 4 weeks, with some cellular invasions into osteoid

In addition, the deposition and calcification of new bone matrix were observed along with the absorption of dentin particles by osteoclast around bone implant materials and a considerable amount of osteoid found in the area which was occupied by soft tissue (Fig. 5).

### Histomorphometric observation

**Fig. 6:**
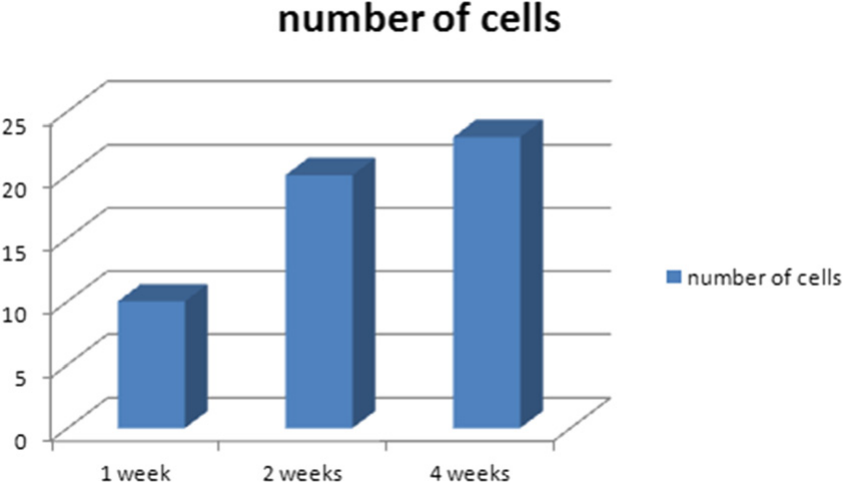
The number of cells attached to the surface of each particle calculated at 1, 2, and 4 weeks

**Fig. 7:**
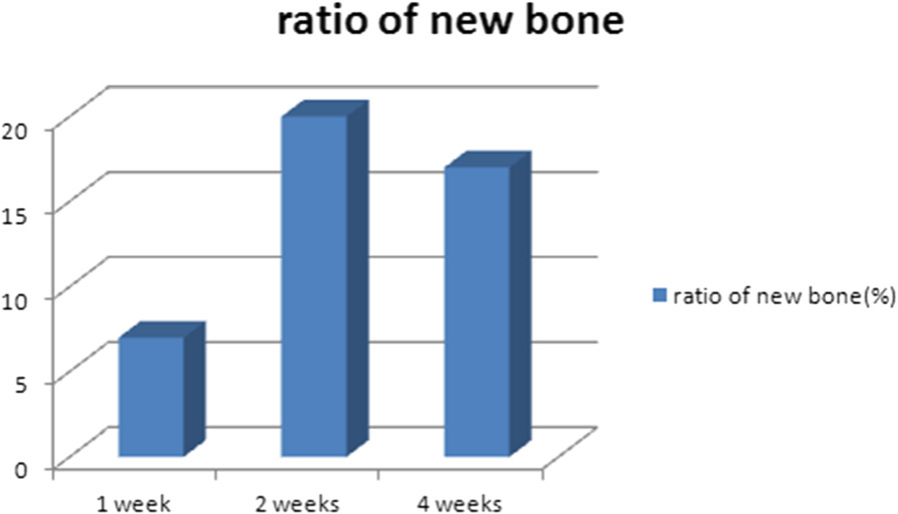
Histomorphometric measurements of newly deposited osteoid and bone on the surface of dentin particles at 1, 2, and 4 weeks

The total number of cells (osteoblast, fibroblast) around DDM particles was divided by the number of particles observed in the samples and then multiplied by 10. With the average value of these being 10, 20, and 23 at 1, 2, and 4 weeks, respectively, a lot of the cells involved in bone induction were formed around particles and increased as time went by (Fig. 6). In addition, the area ratio of new bone was 7, 20, and 17 % at 1, 2, and 4 weeks, respectively, showing the highest value at 2 weeks (Fig. 7).

## Discussion

In this study, by implanting DDM combined with PDRN into the subcutaneous soft tissue of nude mice, we could confirm that bone-forming cells were supplemented and differentiated into osteoblast around graft materials, and then new bone was formed. In other words, we could confirm osteoinduction which induced bone formation. As for the substances showing such osteoinduction, there are bone morphogenetic proteins (BMPs), platelet-derived growth factors (PDGF), insulin-like growth factors (IGF), fibroblast growth factors (FGF), epidermal growth factors (EGF), transforming growth factor or tumor growth factor (TGF), retinoic acid, etc. [9]. Of these, BMP in particular is widely known to induce bone-forming cells when it is implanted into the subcutaneous areas without the bone or under muscles, and its excellent effect in experiments and clinical settings has already been proved in oral and maxillofacial field [10].

Meanwhile, the PDRN used in this study was an activation component which was included in the materials used for healing of cuts and skin regeneration and was composed of parts of low molecular DNA. As purine and pyrimidine nucleotides are formed as a polymer of deoxyribonucleotides which are composed of 50~2000 bases with a combined form via phosphodiester combination, they supply purine and pyrimidine as well as deoxyribonucleotides and deoxyribonucleosides [11]. It is reported that as for PDRN, nucleotides and ne growth of various shapes of cells, synthesis of nucleic acid and healing of cuts [12, 13], salvage pathways to create nucleic acid with low energy consumption [14, 15], and activate A2 purinergic receptors [16]. Also, other reports state that it has an effect on chronic cuts and burns [17, 18].

With many recent studies highlighting the importance of extracellular nucleotides and nucleosides which newly stimulate cell growth, Guizzardi et al. [8] reported that PDRN stimulated fibroblast and collagen by stimulating the aforementioned purinergic receptor system. They evaluated the effect of PDRN in human osteoblast cultured in an experiment and confirmed that PDRN promoted cell growth through the result that a high increase of growth of osteoblast in a PDRN-administered group compared to a control group was seen in their experiment focusing on cell division and alkaline phosphatase activity. In particular, although PDRN-treated cells showed low phosphate activity in the result of the sixth day compared to the control group, alkaline phosphatase activity was shown to gradually increase from the experiment starting day to the 10th day overall, and from these data, PDRN was proved to work as an osteoblast growth stimulant. Sini et al. [6] investigated the effect of Oligo-composite and PDRN on growth and protein secretion of cultured human skin fibroblast and reported that both PDRN and DNAse-treated PDRN promoted the growth of cultured human fibroblast. Also, the cells cultured with PDRN were shown to promote the synthesis and secretion of protein. Thellung et al. [7] confirmed that PDRN and adenosine increased the growth rate of human skin fibroblast in primary culture and reported that PDRN worked as a pro-drug which provided a sufficient amount of mitogenic deoxyribonucleotides, deoxyribonucleosides, and base to cultured cells.

According to these study results, we could confirm that activation of the A2 receptor subtype purinergic receptor through the agency of PDRN promoted propagation of cells and tissue restoration, and it is considered that the function of PDRN as tissue regeneration and medicine is very positive and it can also be applied to the bone defects in the oral and maxillofacial surgical field.

The PDRN used in this study was a material which had been approved and used as a local treatment agent and parenteral medicine in Italy and is known to be effective for leg ulcers, burns, and depressed scars [1]. However, PDRN is liquid phase, and this study needed a scaffold where cells could be attached, grow, and differentiate in the cell differentiation of PDRN to investigate bone-forming capacity, a hard tissue, while previous studies focused on wound healing and regeneration of soft tissue. Therefore, the author, et al., used DDM as a scaffold. As DDM was already proved to have osteoinduction in itself, has little foreign body reaction, and can emit growth factors in a sustained-release (SR) manner due to its porous microstructure. In addition, as it can maintain the shape of an implant site and play the role of structurally reinforcing defects to prevent the twist of neighboring tissue, it can be regarded as an ideal scaffold [2, 3].

The author, et al., could observe bone-forming cells and the expression of new bone through a histological observation of PDRN and DDM implanted into the soft issue of nude mice and looked into the osteoinduction of PDRN and the possibility of DDM as an ideal carrier in a histological and histomorphometric way. In particular, taking a short period, 4 weeks after graft and implant into soft tissue, not hard tissue, into consideration, the hardness of new bone was observed to have advanced considerably. In addition, as the new growing blood vessels entered, absorption of DDM particles were observed, and so it can be considered that DDM plays the role of an ideal scaffold as DDM is slowly absorbed, maintaining the space, and replaced by new bone.

However, the limitations present in this study include failures to verify statistical significance due to a lack of samples in the histomorphometric analysis and to set control group as carrier.

## Conclusions

In this study, an experiment was conducted to verify the bone-forming capacity of PDRN with DDM, a material manufactured with demineralized dentin matrix, after removing impurities from the extracted human teeth, and the following results were obtained. In the result of implanting PDRN and DDM into the subcutaneous area of nude mice, fibroblast, osteoblast, and new bone were induced. In the histomorphometric result, bone-forming cells were observed at 1, 2, and 4 weeks and the formation of osteoinduction cells increased as time went by. In the histomorphometric result, new bone formation was confirmed. Among 1, 2, and 4 weeks, the new bone formation rate was investigated to be highest at 2 weeks.

In conclusion, it was determined that DDM had excellent osteoinduction when it was implanted together with PDRN into the subcutaneous area of nude mice.

## Abbreviations

BMP: bone morphogenetic protein
DDM: demineralized dentin matrix
EGF: epidermal growth factor
FGF: fibroblast growth factor
IGF: insulin-like growth factor
PDGF: platelet-derived growth factor
PDRN: polydeoxyribonucleotide
TGF: transforming growth factor or tumor growth factor
